# Editorial: Moyamoya disease – natural history and therapeutic challenges

**DOI:** 10.3389/fneur.2023.1270197

**Published:** 2023-09-05

**Authors:** Teodor Svedung Wettervik, Markus Fahlström, Johan Wikström, Per Enblad, Anders Lewén

**Affiliations:** ^1^Department of Medical Sciences, Section of Neurosurgery, Uppsala University, Uppsala, Sweden; ^2^Department of Surgical Sciences, Section of Neuroradiology, Uppsala University, Uppsala, Sweden

**Keywords:** moyamoya disease, cerebral blood flow, revascularization, imaging, stroke

Moyamoya disease (MMD) is characterized by gradual stenosis of the terminal internal carotid artery with the development of fragile collateral vessels that partially restore the distal blood flow. These patients are at high risk of developing brain ischemia because of stenosis and intracranial hemorrhage (ICH) due to rupture of the fragile vessels (1). Since MMD is a rare disorder (2), the level of evidence regarding epidemiology, natural history, pathophysiology, imaging, and treatments is limited (1). In this Research Topic; “*Moyamoya disease – natural history and therapeutic challenges”*, we have received several contributions that have brought new insights into many different clinical domains of MMD.

The natural history of MMD remains elusive, but is important to make a prognosis and to determine the optimal timing for interventions. In this Research Topic, Cao et al. conducted a systematic review and meta-analysis on the predictors of MMD disease progression. The authors found younger age, family history, and contralateral vessel abnormalities to be risk factors. It was also interesting to read the study by Liu E. et al., who investigated the role of systemic inflammatory biomarkers, particularly certain blood cell ratios, in MMD. The authors found that patients with MMD exhibited pathological elevations in these biomarkers, but also that the level of elevation differed depending on the disease stage. Thus, biomarkers may play a role in improving the prognostication of MMD.

Furthermore, radiological imaging plays a major part in the diagnosis and subsequent follow-up of MMD. Positron emission tomography-computed tomography (PET-CT), often in conjunction with acetazolamide injection, has been the traditional method to study cerebral hemodynamics (3, 4), but other methods exist, such as arterial spin labeling magnetic resonance imaging (ASL-MRI) (5). In a study by our group, we investigated the potential role of evaluating cerebral hemodynamics with ASL-MRI with an acetazolamide test and dynamic susceptibility contrast MR perfusion (Svedung Wettervik et al.). We found several strengths with the latter method, as it is radiation-free and provided unique information on capillary blood flow, which appeared pathological when the cerebrovascular reserve was exhausted. In another study (Fahlström et al.), we investigated the role of the ASL-MRI-derived coefficient of variation (CoV_cbf_), skewness, and kurtosis, which have previously been associated with cerebral hemodynamics in other conditions (6). However, in the current MMD study (Fahlström et al.), these variables did not carry any hemodynamically relevant information.

Surgical treatment is warranted in MMD patients with exhausted cerebrovascular reserve (4). In a study by Chen et al., combined surgery with direct (superior temporal artery [STA] by-pass) and indirect (encephalo-dura-myosynangiosus) revascularization was often successful. The authors also found postoperative ultrasound examinations of the STA useful as an indicator of the success of indirect revascularization and induced neoangiogenesis. Furthermore, patients with MMD occasionally develop ICHs, which may in some cases be caused by a ruptured MMD-associated aneurysm. These aneurysms are often challenging to treat due to their small size and deep location. In this Research Topic, Zhou et al. present a series of 11 patients with ruptured MMD aneurysms that required endovascular parent-artery occlusion by means of coiling or Onyx. In their study, such treatment usually obliterated the aneurysm, and severe neurological complications were infrequent, suggesting that this approach may be an acceptable rescue therapy when isolated aneurysm occlusion is not feasible. In addition to surgical treatments, certain medications may be beneficial in MMD, e.g., antiplatelets to counteract the formation of intraluminal thrombosis in the pathological MMD vessels. In the systematic review and meta-analysis on antiplatelets in MMD by Liu T. et al., the authors found some support for such agents to decrease mortality and hemorrhagic (but not ischemic) stroke, while it also appeared to increase the by-pass patency following direct revascularization.

Altogether, we have received many important contributions in several different clinical MMD areas. While these bring us further insights into the natural history, adjunct diagnostic/follow-up modalities, and therapeutical options, the rarity of this disease contributes to great challenges in providing reliable answers to clinically important research questions. In future efforts, we need to collaborate to gather “big data” and proceed with clinical trials to find optimal pharmacological agents and to refine the timing and choice of surgical treatment.

## Author contributions

TS: Writing—original draft. MF: Writing—review and editing. JW: Writing—review and editing. PE: Writing—review and editing. AL: Writing—review and editing.
